# Robust and scalable barcoding for massively parallel long-read sequencing

**DOI:** 10.1038/s41598-022-11656-0

**Published:** 2022-05-10

**Authors:** Joaquín Ezpeleta, Ignacio Garcia Labari, Gabriela Vanina Villanova, Pilar Bulacio, Sofía Lavista-Llanos, Victoria Posner, Flavia Krsticevic, Silvia Arranz, Elizabeth Tapia

**Affiliations:** 1 Centro Internacional Franco Argentino de Ciencias de la Información y de Sistemas, Rosario, Argentina; 2 Facultad de Ciencias Exactas, Ingeniería y Agrimensura, Universidad Nacional de Rosario, Rosario, Argentina; 3 Consejo Nacional de Investigaciones Científicas y Técnicas, Rosario, Argentina; 4 Laboratorio Mixto de Biotecnología Acuática, Facultad de Ciencias Bioquímicas y Farmacéuticas, Universidad Nacional de Rosario - Centro Científico Tecnológico y Educativo Acuario del Río Paraná, Rosario, Argentina; 5 Robert H Smith Faculty of Agriculture, Food and Environment, The Hebrew University of Jerusalem, Jerusalem, Israel

**Keywords:** Computational biology and bioinformatics, Molecular biology

## Abstract

Nucleic-acid barcoding is an enabling technique for many applications, but its use remains limited in emerging long-read sequencing technologies with intrinsically low raw accuracy. Here, we apply so-called NS-watermark barcodes, whose error correction capability was previously validated in silico, in a proof of concept where we synthesize 3840 NS-watermark barcodes and use them to asymmetrically tag and simultaneously sequence amplicons from two evolutionarily distant species (namely *Bordetella pertussis* and *Drosophila mojavensis*) on the ONT MinION platform. To our knowledge, this is the largest number of distinct, non-random tags ever sequenced in parallel and the first report of microarray-based synthesis as a source for large oligonucleotide pools for barcoding. We recovered the identity of more than 86% of the barcodes, with a crosstalk rate of 0.17% (i.e., one misassignment every 584 reads). This falls in the range of the index hopping rate of established, high-accuracy Illumina sequencing, despite the increased number of tags and the relatively low accuracy of both microarray-based synthesis and long-read sequencing. The robustness of NS-watermark barcodes, together with their scalable design and compatibility with low-cost massive synthesis, makes them promising for present and future sequencing applications requiring massive labeling, such as long-read single-cell RNA-Seq.

## Introduction

Labelling of nucleic acids using DNA barcodes makes it possible to share the otherwise excessive throughput of next-generation sequencing runs between multiple samples. This is usually achieved by means of individual reactions wherein each sample is tagged with a distinct, known DNA barcode, typically via ligation or tailing PCR. As sequencing throughput continues to increase, however, individual pipetting quickly becomes unwieldy, particularly beyond the numbers that can be handled in one or a few 96-well plates.

In applications where only discrimination between samples is needed, rather than a specific barcode-sample mapping, random-sequence oligonucleotides (randomers) provide a way to dramatically reduce synthesis cost and simplify handling. These have been successfully used in high-accuracy short-read Illumina sequencing, e.g. as unique molecular identifiers (UMIs). However, the use of randomers implicitly requires that the sequencing error rate be sufficiently low, which precludes or significantly impairs their use in more error-prone long-read sequencing platforms. For example, the probability that a 12-mer will be sequenced error free is about 99% for a per-base error rate of 0.1% (representative of Illumina sequencing), but only about 28% for an error rate of 10% (representative of long-read sequencing technologies). Long-read sequencers, such as the MinION device from Oxford Nanopore Technologies (ONT) used in the present study, sequence individual nucleic acid molecules. This results in a low signal intensity which, in turn, translates into reduced raw accuracy, i.e. reads with a high native per-base error rate. Importantly, due to the absence of a specific timing signal or discrete cycles, long-read sequencers produce insertion and deletion errors (spurious additional or missing bases relative to the actual sequence), unlike traditional sequencers, which are only subject to substitution errors (single-nucleotide mismatches).

In order to successfully resolve the barcode identity in spite of such reduced raw accuracy and more complex error profile without a major drop in effective throughput, non-random barcodes with error-correction capacity would be required. However, large sets of error-correcting barcodes present two key challenges: designing good sets of numerous barcodes that are unlikely to be confused one for another and synthesizing such numerous barcodes.

Several authors^1–3^ early recognized that the former challenge is analogous to the issue of reliable transmission of information in the presence of noise. This a well-studied problem in the field of Information Theory^4^, where different approaches have been proposed. In a naive, *non-systematic* approach, large sets of random DNA sequences are generated and screened for a minimum (string) pairwise distance *d*, such that, in the event of sequencing errors, they will remain sufficiently separated to allow their correct identification. The choice of the distance metric is determined by the type of sequencing error. If only substitution errors are expected, the Hamming distance is normally used^5^. If indel errors are additionally expected, the Levenshtein^6^ distance may be used instead. Ideally, to ensure the correct recovery of barcode identities, *d* is constrained to satisfy a proportional relationship with the number of errors *t* expected per barcode sequence (i.e., $d \ge 2 \times t + 1$). This explains why highly error-prone long-read sequencing technologies require barcode sets with large values of *d*. To obtain such barcode sets, in turn, their sequences need a rather large size *l* (typically >24 nt). However, extensive scanning of candidate barcode sequences entails computing pairwise distances with quadratic time complexity^7^ ($l^2$) in search spaces of exponential size ($4^l$). For the purpose of illustration, the size of the search space for $l=36$ (as used in this work) is approximately equal the number of stars in the observable universe. On the other hand, incomplete search results in barcode sets with reduced values of *d*. These, in turn, lead to more reads assigned to erroneous barcodes or “crosstalk”, which is an undesirable effect of sequencing noise.

**Figure 1:**
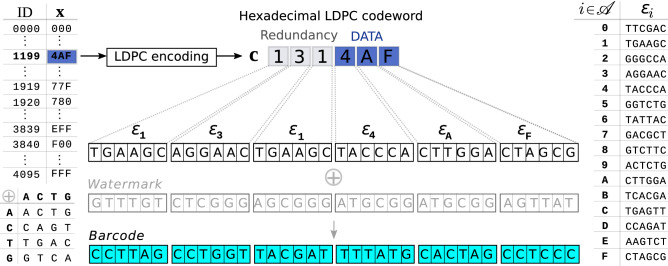
NS-watermark barcodes for long-read sequencing technologies. Each of a set of 4096 IDs is expressed as sequence of $k=3$ symbols from a hexadecimal alphabet $\mathscr {A}$ (**x**, blue). A hexadecimal LDPC code *systematically* adds $m=3$ redundant symbols (grey) to **x** to generate an LDPC codeword **c** of length $n=6$. In the example shown, **x** = 4AF maps to **c** = 131:4AF. A dictionary $\varepsilon$ (right) translates symbols from $\mathscr {A}$ into unique short nucleotide sequences of size $u=6$, thereby converting **c** into a nucleotide sequence of size $l=n \times u=36$. The Galois field addition $\oplus$ of an optional “watermark” sequence of size *l* produces the final target barcode sequence (cyan). The operator $\oplus$ is defined on the table on the bottom left.

In previous work, some of us introduced non-sparse (NS) watermark barcodes^8^, a class of barcodes that instead follows a *systematic* design. NS-watermark barcodes are inspired by watermark error-correcting codes^9^, originally developed to deal with analogous errors in digital communications. NS-watermark barcodes are derived from finite sequences of length *n* defined over a Galois field^10^ of order *q*. Briefly, a Galois field of order *q* is as an alphabet $\mathscr {A}$ of *q* different symbols with specific rules for addition and multiplication. *q* is constrained to be a prime power (i.e. an arbitrary power of a prime number). In the barcode set described in the present work, for example, $q=16=2^4$, which means $\mathscr {A}$ has 16 symbols (i.e. it is a hexadecimal alphabet). Galois fields provide the mathematical basis for the systematic design of sets of sequences with well-established error-correcting capabilities, known as codes. Each sequence of a code (known as a codeword) comprises a payload of useful data (e.g., a sample identifier) and redundancy introduced by the given coding scheme in order to protect said data (Fig. 1) against the harmful effects of noise (e.g., sequencing errors). Low-density parity-check (LDPC) codes^11^ defined on Galois fields of order *q* are amongst the most effective codes against random $q-$ary substitution errors.

Remarkably, knowing how to correct random substitution errors with $q-$ary LDPC codewords is sufficient to correct random substitution and indel errors occurring in closely related quaternary sequences derived from them. For this purpose, we define a dictionary $\varepsilon$ that specifies a mapping from $q-$ary symbols (e.g., symbols in a hexadecimal alphabet $\mathscr {A}$) to short quaternary sequences of size *u*. Using $\varepsilon$, the $q-$ary LDPC codewords of size *n* are translated into nucleotide sequences of size $l = n \times u$. A fixed nucleotide sequence ***w*** of the same size, known as watermark, can optionally be added (Galois-field addition, extended with certain abuse of notation to the addition of elements A/C/T/G in the nucleotide space) to the resulting nucleotide sequences. After tagging and sequencing, barcode sequences appear embedded in continuous nucleotide streams. At this level, random substitutions and indel sequencing errors corrupt barcode sequences with bursts of nucleotide substitution errors that are difficult to overcome. However, at the upper level of $q-$ary symbols, these errors appear as mild substitution errors that the powerful LDPC iterative decoding algorithm^12^ can correct.

If *k* of the *n* *q*-ary symbols in a code carry information, the total number of codewords (and, ultimately, barcode sequences) that can be produced is $q^k$, since each of the *k* symbols can take one of *q* different values. Thus, larger sets of barcodes can be obtained by increasing the number of information-carrying symbols *k*, the alphabet size *q*, or both. Naturally, this will come at the expense of a reduced error-correcting capacity unless the total length *l* is also increased, but an arbitrary number of barcode sequences are possible in theory. In practice, however, a major bottleneck is given by oligonucleotide synthesis cost. Conventional column-based synthesis of the 3840 different oligonucleotides used in this study, for example, would be prohibitively costly. Moreover, individual handling of these oligonucleotides would come at significant additional cost and effort.

To circumvent these limitations and, at the same time, provide error-correction capacity to tolerate the increased error rate of long-read sequencers, we consider a previously unexplored approach. Namely, we evaluate microarray-based synthesis as a source for large non-random oligonucleotide libraries. Specifically, we use 3840 microarray-derived oligonucleotides commercially available as a single pool (OligoMix, LC Sciences). This reduces the synthesis cost by about 40-fold relative to column-based synthesis.

Here, we demonstrate for the first time the general viability of microarray-synthesized oligonucleotide pools as a source for massive error-correcting DNA barcode sets. We also provide an experimental validation of NS-watermark barcodes in particular on the MinION sequencing platform. Finally, we present novel, affordable and low-complexity molecular biology methods for amplification of large oligonucleotide pools and their use for PCR barcoding.

## Results

**Figure 2:**
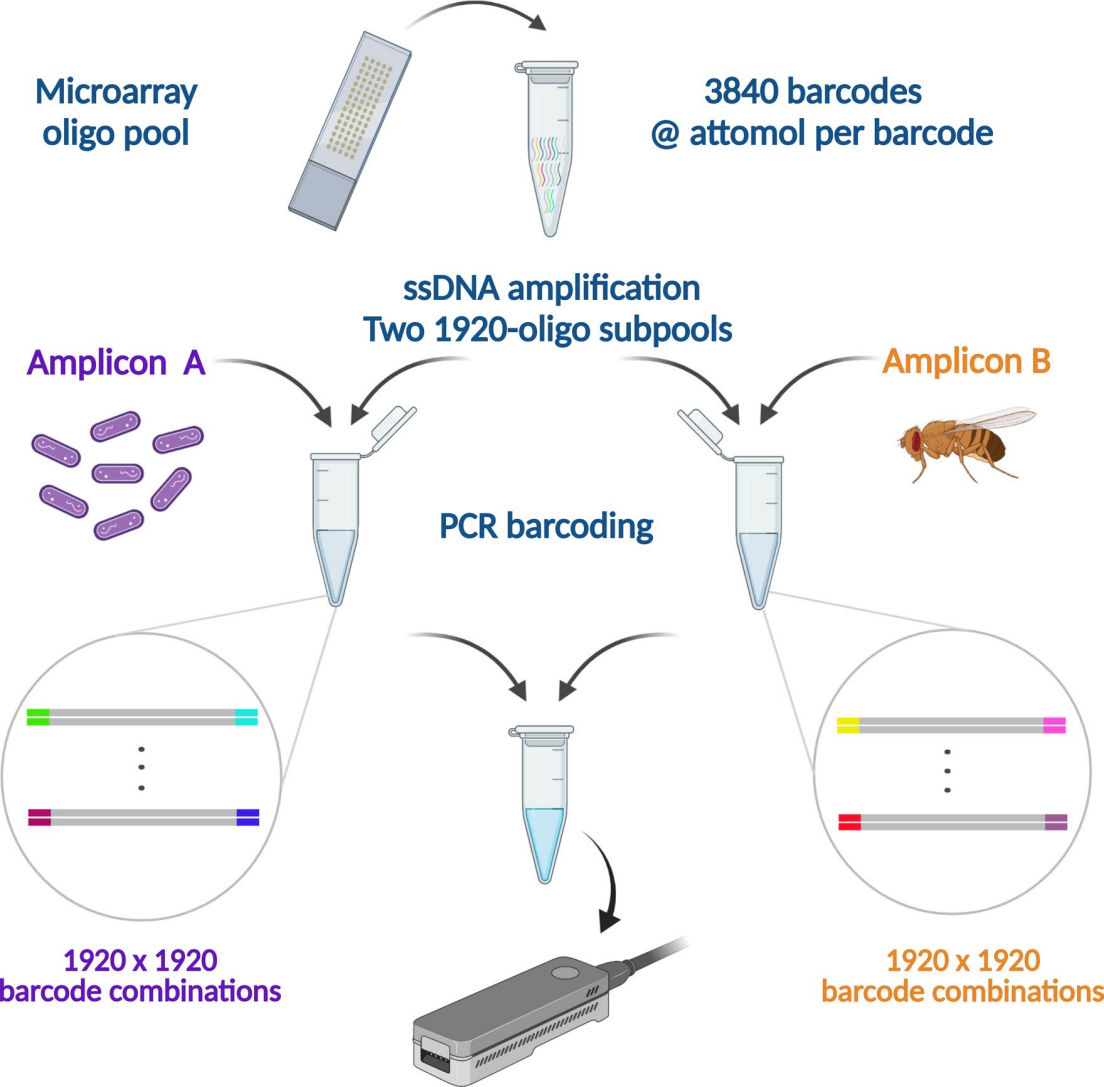
A barnyard experiment (two species) is used to assess the feasibility and performance of NS-watermark barcoding for massively parallel sequencing. A subset of 3840 NS-watermark barcodes, from a set of 4096, is synthesized in a microarray and cleaved to form an oligo pool, which is further divided into two subpools of 1920 barcodes each, followed by preferential amplification of one strand. The resulting ssDNA-enriched subpools are used to tag *B. Pertussis* (sample A) and *D. mojavensis* (sample B) amplicons. Barcoded amplicon subpools A and B are pooled in a molar ratio of 1:2 and sequenced together on the MinION sequencing platform.

In order to assess the feasibility of using microarray-synthesized barcodes for molecular tagging, as well as the overall performance of the NS-watermark design approach previously described by some of us^8^ in the presence of a typical long-read sequencing error profile, we carried out a *proof-of-concept* experiment (Fig. 2). Specifically, we designed a set of 4096 NS-watermark barcode sequences of size $l=36$ nt. We then synthesized 3840 distinct DNA oligonucleotides, chosen at random from the original set, and used them to barcode amplicons from *Bordetella pertussis* (400-bp amplicon, sample A) and *Drosophila mojavensis* (538-bp amplicon, sample B), two evolutionarily distant species (the former prokaryotic and the latter eukaryotic). The species and amplicons have been chosen to have very low sequence identity, such that sequencing reads can be unambiguously mapped to one or the other. Half of the synthesized barcodes (1920), also chosen at random, were used to label molecules from sample A, while the remaining 1920 were used to label molecules from sample B. Each molecule received a random pair of barcodes from the respective pool ($1920 \times 1920$ possible combinations on each pool). The remaining 256 barcodes were not synthesized, but instead used as negative controls, as explained below. Barcoded samples A and B were pooled in a molar ratio of 1:2 and sequenced together on the ONT MinION sequencing platform.

### Affordable and scalable PCR barcoding with thousands of distinct barcodes

Although microarray-based methods provide an attractive alternative to column-based methods for the scalable synthesis of oligonucleotides with non-random sequence, they present two key challenges which have precluded their use for barcoding applications until now. The first is the significantly reduced scale of synthesis, which can be in the order of a few tens of attomoles per oligo, compared to a typical yield of tens of nanomoles or more for column-based synthesis. The second is the fact that microarray-derived oligonucleotides are provided as a single pool and therefore cannot be handled individually. The first challenge can be readily addressed by including primer annealing sites at either end of the oligonucleotides to allow their amplification via PCR, while the second can be partially addressed by defining oligo subpools and making said primer annealing sites subpool-specific. In this work, we flanked the 36-bp variable barcode sequence with two sets of fixed, 20-bp primer annealing regions (BPFA/CSA and BPFB/CSB) which define two subpools of 1920 oligos each (A and B, respectively) and used them to selectively amplify each subpool in two parallel PCR reactions (Supplementary Figure 1, top).

After subpool-specific amplification, a remaining obstacle is that the PCR product is in double stranded form, unsuitable for priming a downstream barcoding reaction. Digestion with a nicking endonuclease^13^ followed by purification via denaturing gel electrophoresis has been described for isolating one strand of similar constructs in fluorescence in situ hybridization (FISH) applications^14^. However, for our purposes, this protocol is relatively time-consuming and expensive. Here, we explore the arguably simpler alternative of using an asymmetric PCR (aPCR) reaction to preferentially amplify one strand rather than purify it. Specifically, we reamplified the subpools in aPCRs where the forward primer (BPFA or BPFB) is an $\sim$33:1 molar excess relative to the reverse primer (Supplementary Figure 1, bottom). In order to match concentration-adjusted melting temperatures ($T_m$)^15^, three extra bases (CGC or GCG) are included immediately downstream of the variable barcode sequence on the microarray-synthesized oligos and the reverse primers for the aPCRs are extended accordingly (rcCSA-GCG or rcCSB-CGC).

Given the above, the microarray-synthesized oligo set comprises 3840 sequences of 79 nt each whose canonical strand is of the form 5′-BPFA/B-BCxxxx-GCG/CGC-CSA/B-3′, where BCxxxx is one of the 3840 barcode sequences. Although the carryover of the background subpool following subpool-specific amplification is expected to be in the order of tens of yoctomoles (or a few hundred molecules) and is not expected to affect the subsequent PCR barcoding reaction, oligos were ordered in their reverse complementary form to aid in the detection of any such carryover. Thus, the final oligos as ordered are of the form 5′-rcCSA/B-CGC/GCG-rcBCxxxx-rcBPFA/B-3′, where the prefix rc indicates the reverse complement.

Following ssDNA enrichment, CSA and CSB (without the extra GCG/CGC) are intended to double as primers for a barcoding PCR, with the remainder of the respective sequence as an overhang (Supplementary Figure 2). Specifically, CSA and CSB anneal to the ends of amplicon samples A and B, previously obtained using target-specific primers tailed on either end with CSA and CSB or the reverse complements thereof, forming inverted terminal repeats (ITR). This particular design makes it possible to tag the amplicons at their 5′ and 3′ ends using the same set of barcode oligos. We evaluated the feasibility of this amplicon design in terms of hairpin loop formation both in silico (using Mfold^16^) and by preliminary amplification essays. For very short templates where hairpin formation is a concern, the ITR configuration may be dropped by using unrelated consensus sequences at either end, at the expense of doubling the number of oligos that need to be synthesized.

As a small but crucial design consideration, a single additional adenine  (A) was included at the 3′ end of the CSA and CSB sequences in primers used for tailed amplicon generation to account for the terminal transferase activity of the *Taq* polymerase used for the previously described ssDNA enrichment aPCRs, since a single mismatch on the 3′ of a PCR primer can have a catastrophic effect on amplification efficiency, particularly for the case of a mismatching adenine^17^. The final primers for tailed amplicon generation are thus of the form 5′-CSA/B-A-FPA/FPB/RPA/RPB-3′, where FPA, RPA are the forward/reverse primers for *B. pertussis* and FPB/RPB are the forward/reverse primers for *D. mojavensis*.

Although the tailed amplicon generation and PCR barcoding reactions have been described separately above for the purpose of clarity, in practice they can be (and were) combined into a single reaction in order to avoid purifying and/or concentrating ssDNA product. In any case, the barcoded products for pools A and B were pooled in a molar ratio of 1:2 (A:B) and subject to standard library preparation.

### MinION sequencing and raw output analysis

**Figure 3:**
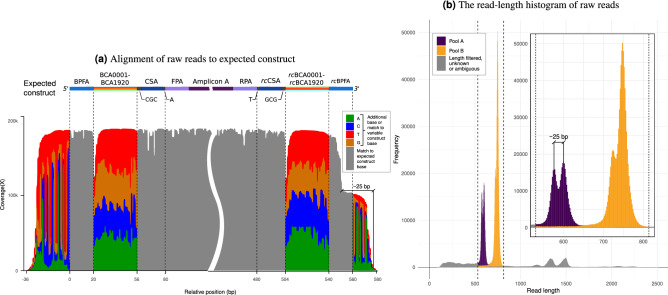
Analysis of raw sequencing output. (**a**) Alignment of raw reads to the expected barcoded construct for amplicon A, which includes forward and reverse species-specific primers (FPA/RPA), along with a highly-variable barcode region, flanking sequences CSA and BPFA, and auxiliary sequences CGC and A, or the reverse complement (rc) thereof. Only reads aligning in the forward sense are shown to highlight that about half the reads are trimmed ~25 bp shorter at the end. (**b**) Read-length histogram of raw reads with ground truth assignment to pools A or B. Dotted lines show the window used for length filtering. Reads outside the window or with either unknown or ambiguous ground truth were not considered and are shown in grey. **(b-inset)** Zoom in on the valid length window, showing secondary peaks (bimodality) due to imperfect adapter trimming.

The prepared library was sequenced on a MinION device for 14.5 hours, after which the data were basecalled and 1.6 million “pass” quality reads were obtained. Alignment of the raw pass reads to the expected construct (known *B. pertussis* and *D. mojavensis* sequence flanked by auxiliary sequences) confirmed successful barcoding and well-formed molecules, including hyper-variability of the barcode region (Fig. 3a).

The alignments also confirmed a ratio of A to B reads close to the expected 1:2 and showed additional sequence on either end, corresponding to sequencing adapters. Further, sense-specific alignment revealed an artifact whereby approximately half of the reads for pool A (or about one third for pool B) were trimmed about 25 bp shorter at the end. This is in agreement with the observed read-length histogram, which shows two pairs of peaks centered at 590 and 740 bp, right-shifted relative to the nominal amplicon sizes of 560 and 698 bp (Fig. 3b). We defined a window of $\pm 10\%$ around the peak pair centers ($590 - 10\%$ to $740 + 10\%$), which accounts for the maximum expected negative or positive change in read length due to random insertion and deletion errors (approximately 3 standard deviations). Reads outside this window were excluded from downstream processing, including the calculation of performance metrics. This length filtering eliminates chimeric read peaks at double/triple amplicon size, the DNA control strand used in ONT sequencing and background short reads, all of which are not only outside the control of the barcoding system but also of little to no practical importance. About 25% of total reads are discarded this way (Supplementary Figure 3).

Reads surviving length filtering were classified as belonging to amplicon samples A or B if they uniquely mapped along most of their length to the known *B. pertussis* or *D. mojavensis* sequences, respectively. This was taken as “ground truth” given the low identity (and consequently low probability of spurious alignments) between the two sequences/species. Reads which did not map or did so ambiguously or incompletely were excluded from further processing. This second filtering step, which removes less than 1% of total reads, focused on the removal of residual chimeric or off-target reads.

### Barcode demultiplexing and performance metrics

Since barcode boundaries may shift relative to their expected (design) positions due to drift caused by sequencing errors and inexact read start/end positions, as evidenced by variable and bimodal read lengths, we leverage the presence of the consensus sequence (CSA/B) and primers (FPA/B and RPA/B) to detect actual barcode positions. Specifically, in a forward-sense match, we expect the barcode sequence to be immediately upstream of CSA/B. Hence, we first align the CS+FP/RP region against each read to be demultiplexed. Only four alignments are needed per read (forward/reverse primers for A/B, plus auxiliary sequences), regardless of the number of barcodes. For each alignment above a certain quality threshold, we extract a subread consisting of a window around the identified barcode end position. The approach for a reverse-sense match is analogous, except subreads must be reverse complemented before being fed to the decoder. These alignments are also used to locally estimate the substitution, insertion and deletion probabilities by computing the average observed frequency of each event from the alignment CIGAR string. These probability estimates are needed by the decoder.

Subreads prepared according to the above are demultiplexed using a *q*-ary LDPC iterative decoder algorithm in conjunction with a nucleotide-level Hidden Markov Model (HMM) predictor of *q*-ary symbol posterior probabilities, as previously described^8^. Briefly, this two level decoder confirms global synchronization by “walking” upstream along the consensus sequence and primer (i.e. performing a backward pass along CGC/GCG-CSA/B-A-FPA/B or CGC/GCG-CSA/B-A-RPA/B in the sense of Davey and MacKay^9^); transforms insertion, deletion and substitution errors into equivalent probabilistic (soft) substitution errors by applying a so-called forward-backward algorithm; and decodes the barcode in the *q*-ary domain through an iterative decoding algorithm. The output of the decoder is either one of the 4096 barcode identities (including the 256 negative controls) or a flag indicating that the barcode failed to be decoded, in which case the subread is discarded.

To supplement this decision, we introduce a decoding confidence metric *L* which estimates the likelihood that the output identity is correct. Subreads whose confidence value is below a certain threshold can be voluntarily discarded, which enables explicit control of the trade-off between two key performance metrics: the read recovery rate and the crosstalk rate. The read recovery rate is defined simply as the percentage of considered subreads which are not discarded either by the decoder, voluntarily due to low confidence or due to being decoded as a negative control. The crosstalk rate is defined as the percentage of recovered subreads which are assigned to an erroneous barcode.

Since the 1920 barcodes within each subpool were used here to tag the same amplicon, alignment-based ground truth can only reveal inter-subpool decoding errors, while intra-subpool errors are not directly detectable. However, under the uniformity assumption that mistaking any barcode for any other is equally likely, undetectable intra-subpool errors (A mistaken for another A or B mistaken for another B) can be estimated as equal to inter-subpool errors (A mistaken for B or B mistaken for A, respectively).

In a real-life scenario where ground truth information is unavailable altogether, the crosstalk rate can be alternatively estimated as proportional to the percentage of reads that decode to a negative control, where the factor of proportionality accounts for the difference in subset sizes (in this case, $\frac{3840}{256}$)^18^. This also holds only under the above uniformity assumption. For the more general case of non-uniformity, the estimation based on negative controls is biased, but the bias may be approximately corrected by explicitly considering the pairwise edit distances between barcodes and their non-uniform concentrations at the time of tagging (Supplementary Information Section 3).

### Demultiplexing performance

**Figure 4:**
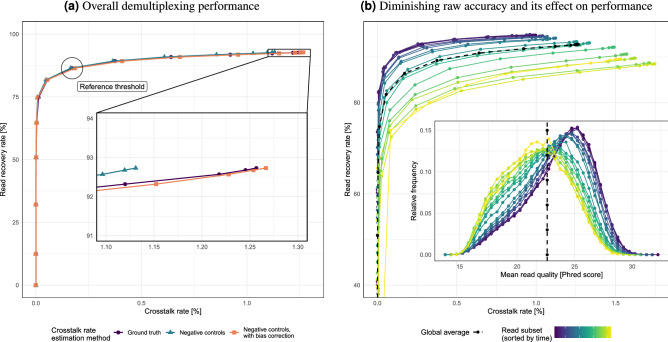
Demultiplexing performance of NS-watermark barcodes. (**a**) A variable threshold tunes the trade-off between the read recovery and crosstalk rates. An arbitrary threshold corresponding to 86.4% recovery and 0.17% crosstalk is highlighted for the purposes of discussion. **(a-inset)** Crosstalk estimated based on the set of 256 negative controls (teal) slightly underestimates the crosstalk rate based on ground truth (purple), but this bias can be approximately corrected (orange). (**b**) Reads are divided in subsets of 100,000 (sorted by time) to show that mean raw read quality diminishes as sequencing progresses (0–14.5 h). Thus, demultiplexing of young (purple) or old (yellow) subsets performs better or worse, respectively, than average (dashed, black).

Based on the above, we analyzed the trade-off between the crosstalk rate and the read recovery rate at increasingly stringent thresholds for the decoding confidence metric (Fig. 4a). At an arbitrary reference point at the “knee” of the demultiplexing performance curve, we report a read recovery rate of 86.4% at a crosstalk rate of 0.17% ($\approx$ 1 misassignment for every 584 demultiplexed reads), which correspond to 86.25% recall and 99.83% precision in terms of the metrics used in a benchmark ONT demultiplexing study with small barcode sets^19^. However, as noted above, other combinations can be achieved by varying the confidence threshold, such as 90.9% recovery at 0.64% crosstalk, 74.7% recovery at 0.01% crosstalk, or 50.9% recovery at 0.001% crosstalk (1 misassignment for every 156, 10,000 or 100,000 reads, respectively).

We then analyzed the impact of sequencing quality on the demultiplexing performance. As previously reported^20^, the quality of nanopore sequencing reads degrades significantly over time. Naturally, this negatively impacts demultiplexing performance (Fig. 4b). Specifically, the read recovery rate for a given crosstalk rate degrades by about 10-15 percentage points from the best to the worst condition. However, the performance degradation observed is smooth, with no steep decline at any point during the extended sequencing time (14.5 h).

Finally, we report full representation of the microarray-synthesized oligonucleotides: all 3840 barcodes in subpools A and B were detected in at least 10 of the reads, while none of the 256 negative control barcodes was detected in more than 5 reads (Supplementary Figure 5-inset). Similarly, rich diversity was observed in terms of barcode combinations. In the ~0.9 million reads (~1.8 million subreads) with successfully demultiplexed, matching barcodes on either end (A-A or B-B), we observed ~0.8 million distinct barcode combinations. Of these, ~0.7 million (88.6%) combinations were unique, ~0.08 million (9.9%) appeared twice, ~10,000 appeared thrice (1.2%) and none appeared more than 16 times.

## Discussion

Here, we evaluated the feasibility of microarray-synthesized NS-watermark barcodes for sequencing on the ONT MinION platform. We were able to demultiplex more than 86% of filtered reads tagged with 3840 barcodes with a crosstalk rate of 0.17%. This level of crosstalk is comparable to that exhibited by 96-plex single-end barcoding schemes in the Illumina platform^21^ despite the higher plexity and the combined error rate of microarray-based synthesis, multiple rounds of PCR amplification using low-fidelity *Taq* polymerase and native, single-pass ONT sequencing.

The number of distinct barcodes synthesized and demultiplexed here is one order of magnitude higher that the largest commercially-available barcoding kits for long-read sequencing (3840 vs. PacBio’s 384), while the diversity afforded by combinatorial asymmetric barcoding with this many distinct tags would be close to that offered by 12-nt unique molecular identifiers or UMIs ($3840 \times 3840 \approx 14.7~\text {M}$ vs. $4^{12} \approx 16.7~\text {M}$). Even higher numbers seem easily within reach, as the NS-watermark barcode design framework is arbitrarily scalable (limited only by barcode length), the time complexity of demultiplexing is constant in the number of barcodes (and linear in barcode length), and microarray-based synthesis of tens of thousands to millions of oligonucleotides per chip is already commercially available.

Slightly longer microarray-synthesized NS-watermark barcodes (or combinatorial tagging with more than two of the current ones, e.g. by ligation) could, in principle, provide sufficient diversity for use as non-random UMIs, even in multiplex, long-read applications. Such non-random UMIs would require no clustering, since the universe of valid UMIs would be known. Similarly, there would be no need for hybrid (long- and short-read) sequencing pipelines^22^, multiple passes of the UMI sequence^23,24^, non-standard synthesis chemistry^25^ and other similar approaches which ultimately derive from the lack of error correction capacity of random tags traditionally used for this purpose.

In view of the above, NS-watermark barcodes, particularly when combined with scalable, low-cost microarray-based synthesis, are a promising tool for long-read applications which require massive barcoding, high read recovery and low crosstalk.

One remaining concern is that the exponential amplification during PCR and aPCR exacerbates initial manufacturing differences in concentration between individual barcodes within each subpool. This results in significant non-equimolarity after amplification (Supplementary Figure 5), which may be particularly relevant for quantitative barcoding applications. The issue could be partially alleviated by performing a single asymmetric PCR reaction directly on the raw oligo pool (rather than performing two rounds of PCR, as described here). It should also be noted that the use of combinatorial barcoding reduces the impact of non-equimolarity simply by expanding the universe of possible values, such that repeated values are less likely to occur. Here, for example, although individual barcode concentrations varied by about two orders of magnitude, most (88.6%) barcode combinations were unique and none of them were heavily over-represented (maximum frequency of 16).

## Methods

### Tailed amplification of DNA fragments

DNA fragments of *B. pertussis* and *D. mojavensis*, corresponding to 400 bp of the repeated insertion sequence IS-481^26^ and 538 bp of the Ppr-Y gene, respectively, were amplified by PCR using tailed species-specific primers (Supplementary Information Table S1). Specifically, previously described^27^ forward (FPA) and reverse (RPA) primers tailed with the consensus sequence CSA 5′-GCAAGCGGTACACTCAGATC-3′ plus a single adenine were used for the *B. pertussis* fragment (CSA-A-FPA 5′-GCAAGCGGTACACTCAGATC**A** GACTTCGTCTTCGTGGCCAT-3′, CSA-A-RPA 5′-GCAAGCGG TACACTCAGATC**A** GTACAGCGCGCCCGATGCCT-3′). Similarly, specific forward (FPB) and reverse (RPB) primers tailed with the consensus sequence CSB 5′-CAGGAGTTGTCTAGGCGATC-3′ plus a single adenine were used for the *D. mojavensis* fragment (CSB-A-FPB 5′-CAGGAGTTGTCTAGGCGATC**A** CGAGTATCTTCAAGAAAAAGAAATTCAA-3′, CSB-A-RPB 5′-CAGGAGTTGTCTAGGCGATC**A** CGTGTAAATGCAATTCCTGAGACAT-3′).

The PCR reactions were performed in a volume of 25 $\upmu \hbox {L}$ containing 1X *Taq* Polymerase Buffer (Invitrogen), 1.5 mM $\hbox {MgCl}_{2}$, 0.4 $\upmu \hbox {M}$ pool-specific forward primer (CSA-FPA or CSB-FPB), 0.4 $\upmu \hbox {M}$ pool-specific reverse primer (CSA-RPA or CSB-RPB), 1 U of *Taq* Polymerase (Invitrogen), 0.2 mM each dNTP, and approximately 7 ng of *B. pertussis* or *D. mojavensis* DNA fragments. Amplifications were carried out in a Verity Fast Thermal cycler (Applied Biosystems) using a touchdown thermal profile consisting of two cycles of $95^\circ \hbox {C}$ for 30 s, $56^\circ \hbox {C}$ for 45 s, and $72^\circ \hbox {C}$ for 2 min, two cycles of $95^\circ \hbox {C}$ for 30 s, $55^\circ \hbox {C}$ for 45 s, and $72^\circ \hbox {C}$ for 2 min, 30 cycles of $95^\circ \hbox {C}$ for 30 s, $54^\circ \hbox {C}$ for 45 s, and $72^\circ \hbox {C}$ for 1 min, and a final extension step of $72^\circ \hbox {C}$ for 5 min. PCR products were evaluated on a 2% agarose gel stained with GelGreen (Biotium, USA).

### Oligo pool design and the selective amplification of oligo sub pools for PCR barcoding

A pool of 3840 different 79-nt DNA oligonucleotide sequences (OligoMix) was ordered from LC Sciences, LLC (Houston, Texas, USA). Each oligo included a 36-nt barcode sequence (one of BCA0001 to BCA1920 for pool A; one of BCB0001 to BCB1920 for pool B) flanked by two subpool-specific primer annealing sites: BPFA and CSA for pool A; BPFB and CSB for pool B. Sequences in the oligo pool were requested in their reverse complementary form (Supplementary Information Table S1). The oligo pool was amplified in two independent PCR reactions using a pair of subpool-specific primers: BPFA and rcCSA for pool A; BPFB and rcCSB for pool B. These PCR reactions were performed in a volume of 50 $\upmu \hbox {L}$ containing 1X *Taq* Polymerase Buffer (Invitrogen), 1.5 mM $\hbox {MgCl}_{2}$, 1 $\upmu \hbox {M}$ forward primer BPFA or BPFB, 1 $\upmu \hbox {M}$ reverse primer rcCSA or rcCSB, 1 U of *Taq* Polymerase (Invitrogen), 0.2 mM each dNTP, and 1 $\upmu \hbox {L}$ of serial dilutions of the OligoMix. Specifically, we tested serial dilutions of 1:1 (no dilution), 1:4, 1:16 and 1:64. The original concentration of the oligo pool was reported by the manufacturer as 2137.5 pg/$\upmu \hbox {L}$. PCR reactions were carried out in a Verity Fast Thermal cycler (Applied Biosystems); the program consisted of $95^\circ \hbox {C}$ for 5 min, three cycles of $95^\circ \hbox {C}$ for 30 s, $50^\circ$ for 30 s, and $72^\circ \hbox {C}$ for 15 s, followed by 40 cycles of $95^\circ \hbox {C}$ for 30 s, $52^\circ \hbox {C}$ for 30 s, and $72^\circ$ for 15 s, and finally $72^\circ \hbox {C}$ for 5 min. An agarose gel stained with GelGreen (Biotium, USA) was used to verify PCR products and select the best titer (1:64).

Asymmetric PCR reactions were performed in 25 $\upmu \hbox {L}$ containing 1X *Taq* Polymerase Buffer (Invitrogen), 2 mM $\hbox {MgCl}_{2}$, 1 $\upmu \hbox {M}$ forward primer (BPFA or BPFB), 0.03 $\upmu \hbox {M}$ reverse primer (rcCSA-GCG or rcCSB-CGC) (Supplementary Information Table S1), 1 U of *Taq* Polymerase (Invitrogen), 0.26 mM each dNTP, and approximately 7 ng of DNA of the subpool amplification products A or B. Amplifications were carried out in a Verity Fast Thermal cycler (Applied Biosystems); the amplification program consisted of $95^\circ \hbox {C}$ for 3 min, 22 cycles of $95^\circ \hbox {C}$ for 10 s, $52^\circ \hbox {C}$ for 15 s, and $72^\circ \hbox {C}$ for 15 s. After 22 cycles of the asymmetric PCR reactions, the final random dual PCR barcoding of each amplicon sample was accomplished by directly adding 1 $\upmu \hbox {L}$ of a 1:100 dilution of the respective tailed amplicon product, A or B.

The random dual PCR barcoding reactions were then finished with 23 additional cycles of $95^\circ \hbox {C}$ for 30 s, $52^\circ \hbox {C}$ for 30 s, and $72^\circ \hbox {C}$ for 60 s, and finally $72^\circ \hbox {C}$ for 3 min. The presence of 560-bp *B. pertussis* barcoded amplicons and 698-bp *D. mojavensis* barcoded amplicons was confirmed by agarose gel electrophoresis. Final PCR products were further analyzed by Sanger sequencing, which confirmed the size and sequence of the fragments, as well as the presence of a high variability region of 36 bp in length, coinciding with the length of the barcodes. PCR products were purified by carefully mixing 1.8X volume of NucleoMag beads (Macherey-Nagel, Germany) in DNA LoBind tubes followed by 10 min incubation at RT to allow binding. After pelleting on a magnet, and two washes in 200 $\upmu \hbox {L}$ ethanol (80%) with drying at $37^\circ \hbox {C}$, the pellet was eluted in nuclease-free water (NFW), and pelleted again on a magnet. The eluate was transferred to a fresh DNA LoBind tube. Barcoded amplicons from both species were quantified with the Qubit$\circledR$ Fluorometer (Invitrogen) and Qubit$\circledR$ dsDNA BR Assay Kit to check the recommended 1 $\upmu \hbox {g}$ DNA input to the ONT sequencing protocol.

### Sequencing library preparation for MinION sequencing

End-repair and dA tailing were carried out with the NEBNext FFPE Repair Mix (New England BioLabs, USA) and the NEBNext$\circledR$ Ultra II End-Repair/dA-tailing Modules (New England BioLabs, USA). After incubation at $20^\circ \hbox {C}$ for 5 min and $65^\circ \hbox {C}$ for 5 min, the end-repaired and dA-tailed DNA barcoded amplicons from both species were cleaned up with 1.8X volume of NucleoMag beads at RT. The samples were combined in equal volumes, and adapters were ligated using the NEBNext Quick Ligation Module (New England BioLabs, USA) following the standard ONT protocol. This was followed by purification with 0.6X volume of NucleoMag beads and preparation for sequencing according to the standard Oxford Nanopore Technologies protocol (SQK-LSK109). Quantification of 1 $\upmu \hbox {L}$ of the adapted library with the Qubit$\circledR$ fluorimeter showed a DNA concentration of 73.1 ng/$\upmu \hbox {L}$ or, equivalently, 170 fmol/$\upmu \hbox {L}$, sufficient for loading the library onto the MinION flow-cell.

### MinION sequencing, basecalling, and the preprocessing of sequencing reads

A MinION flow-cell (R9.4.1) with 1461 pores available was primed with 800 $\upmu \hbox {L}$ of the Priming Mix via the priming port. After 5 min of incubation, another 200 $\upmu \hbox {L}$ of the Priming Mix were added via the priming port, this time with the SpotOn port opened. A sequencing library mix was prepared with 12 $\upmu \hbox {L}$ of the DNA library, 37.5 $\upmu \hbox {L}$ of Sequencing Buffer (SQB) and 25.5 $\upmu \hbox {L}$ of the Loading Beads (LB) mixed immediately before use. The library was loaded onto the flow-cell and run for 14.5 hours. Raw data was processed with Guppy 4.015 configured to use the high accuracy mode and GPU basecalling, with otherwise default settings. A dataset comprising 1,633,136 “pass” quality reads was obtained. Reads shorter than 531 bp or longer than 814 bp were discarded. The remaining reads were aligned to the target amplicon sequences (400 bp of the *B. Pertussis* repeated insertion sequence IS-481 and 538 bp of the *D. Mojavensis* Ppr-Y gene) using minimap2 with parameters -x map-ont –secondary=no –MD. Resulting alignments were taken as ground truth for the purposes of subpool assignment as long as they were unambiguous (hit to IS-481 or Ppr-Y but not both) and covered the entire length of the read minus at most 200 bp.

### The NS-watermark demultiplexing pipeline

The following sequences and the reverse complements thereof were aligned to each read using the C++ version of edlib^28^ v1.2.7 in HW mode: CCG-CSA-A-FPA, GCG-CSB-A-FPB, CCG-CSA-A-RPA, GCG-CSB-A-RPB. All hits with an edit distance lower than or equal to 12 were kept. Estimates of the substitution, insertion and deletion probabilities $P_s$, $P_i$ and $P_d$ were computed from the CIGAR strings of these alignments. For each forward (resp., reverse) hit, a fixed-length sequence was extracted by defining a window of $-61$ to +56 bp (resp., $-56$ to +61 bp) around the alignment start (resp., end) position. For reverse sense hits, the extracted sequence was reverse complemented. Extracted sequences were decoded using a previously reported^8^ pipeline with $q=16$, $n=6$, $k=3$, $m=n-k=3$, $u=6$ and other parameters as provided in Supplementary Data. Given the decoder output $\varvec{d} = \left( d_1, \ldots , d_n \right)$, a confidence metric was computed as $L\left( \varvec{d}\right) \,=\prod _{\,i=1}^{\,n} {L \left( d_i \right) }$, where $L\left( d_i \right) = P\left( \varvec{r } \, | \, { d_i}\right)$ are the likelihoods of the observed corrupted barcode sequence $\varvec{r}$ given the transmission of *q*-ary symbols $d_i$, $i=1, \ldots , n$, which are obtained as part of the decoding process^8^ (p. 810). If the original alignment had produced more than one forward (or reverse) hit, the one producing the highest *L* was output.

## Supplementary Information

Supplementary Information.

## Data Availability

Sequence data that support the findings of this study have been deposited in Sequence Read Archive (SRA) database and will be accessible through SRA accession number SUB9449266 upon publication. All other relevant data are available from the corresponding author upon reasonable request.
