# Supplementary material for: Robust and scalable barcoding for massively parallel long-read sequencing
Source: Sci Rep. 2022 May 10;12:7619. doi: 10.1038/s41598-022-11656-0 (PMC9090787; doi:10.1038/s41598-022-11656-0)
Supplement: Supplementary file 1 — Supplementary Information. [file 41598_2022_11656_MOESM1_ESM.pdf]

Supplementary Information

Robust and scalable barcoding for massively parallel long-read sequencing

Ezpeleta J.*, Garcia-Labari I., Villanova G. V., Bulacio P., Lavista-Llanos S., Posner V., Krsticevic F., Arranz S. and Tapia E.

*Corresponding author. Email: ezpeleta@cifasis-conicet.gov.ar

1 Random dual barcoding with microarray oligo pools

A set of 3840 barcodes of 79 nt each was synthesized in a microarray oligo pool. Each barcode sequence includes a pair of flanking sequences allowing the differentiation and the amplification of oligo subpools A and B of 1920 barcodes each. Double-stranded DNA molecules from each of the oligo subpools are passed through an asymmetric PCR reaction to obtain designated single-stranded DNA molecules over a background of double and single-stranded DNA molecules (**Supplementary Figure 1**). Single-stranded DNA molecules following the aPCR reactions are ready for performing the random dual barcoding (**Supplementary Figure 2**) of tailed amplicon samples from *Bordetella pertussis* and *Drosophila mojavensis*.

2 Read processing and demultiplexing statistics

A set of 1,633,136 random-dual barcoded reads was obtained and length-filtered to eliminate chimeric and background short reads. The resulting subset of 1,213,131 length-filtered reads was again filtered based on the availability of ground truth molecular identities. The subset of 1,197,177 length-filtered reads with ground truth molecular identities was further processed to extract a set of 2,394,354 single-end barcoded subreads, ready for NS-watermark demultiplexing. Among these reads, 727,318 were from amplicon A and 1,667,036 from amplicon B (close to the expected 1:2 ratio). After decoding, we observed 325,263 discarded subreads and 2001 incorrectly decoded subreads, including 57 (170) amplicon A (B) subreads incorrectly decoded to barcodes in the negative control set of 256 barcodes, as well as 448 (1326) subreads of amplicon A (B) incorrectly decoded to the pool B (A). To complete the picture, we assumed the number of unobservable amplicon A subreads incorrectly decoded to other barcodes in pool A (intra-pool errors) equals the number of subreads of the amplicon A incorrectly decoded to the pool B, namely 448. We made the same assumption about the unobservable number of subreads of the amplicon B incorrectly decoded to the pool B (1326). The assumption seems reasonably since both pools of barcodes A and B have the same number of barcodes (1920) and share similar distributions of pairwise edit distances (**Supplementary Figure** 4). On the whole, these numbers lead to 3775 incorrectly demultiplexed subreads, 645,819 subreads correctly demultiplexed to pool A and 1,419,497 subreads correctly demultiplexed to pool B, for a total of 2,065,316 subreads. As a result

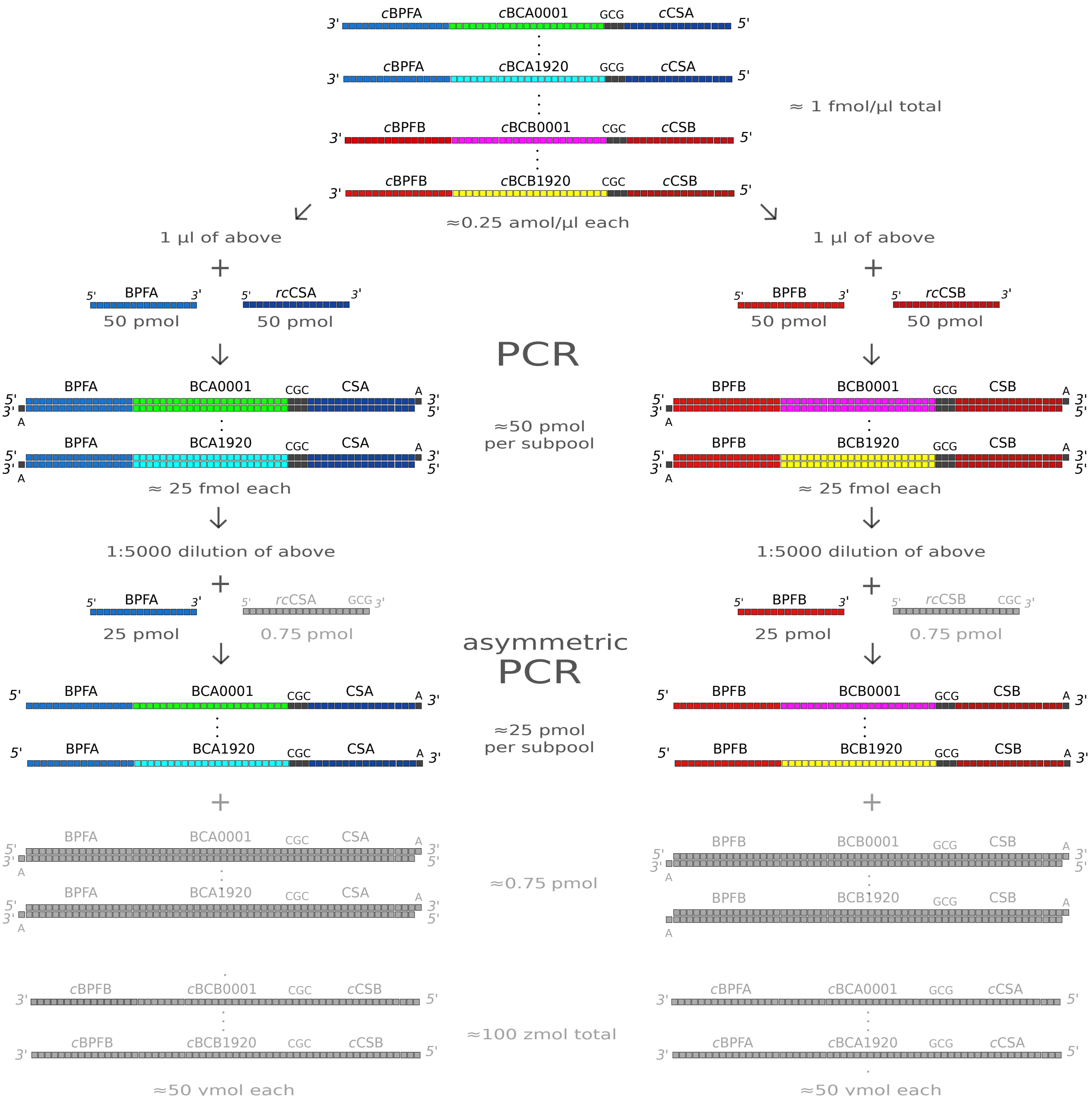

Supplementary Figure 1: Two-round PCR separation and amplification of oligo subpools A and B. A first symmetric PCR selectively amplifies each subpool. A second asymmetric PCR preferentially amplifies the top strand. Except for the initial concentration, which was provided by the manufacturer, quantities shown are nominal and assume unitary reaction efficiencies.

(**Supplementary Figure** 3), we report a subread recovery rate of 86.41% at crosstalk rate of 0.17% or, equivalently, a 86.25% recall with a 99.83% precision.

3 Unbiased estimation of the crosstalk rate

As previously described [1], the expected Frequency of False Assignments (FAF) in a multiplex sequencing experiment involving S barcodes randomly selected from some major *universe* of barcodes comprising C unique members can be estimated from the number of reads F incorrectly demultiplexed

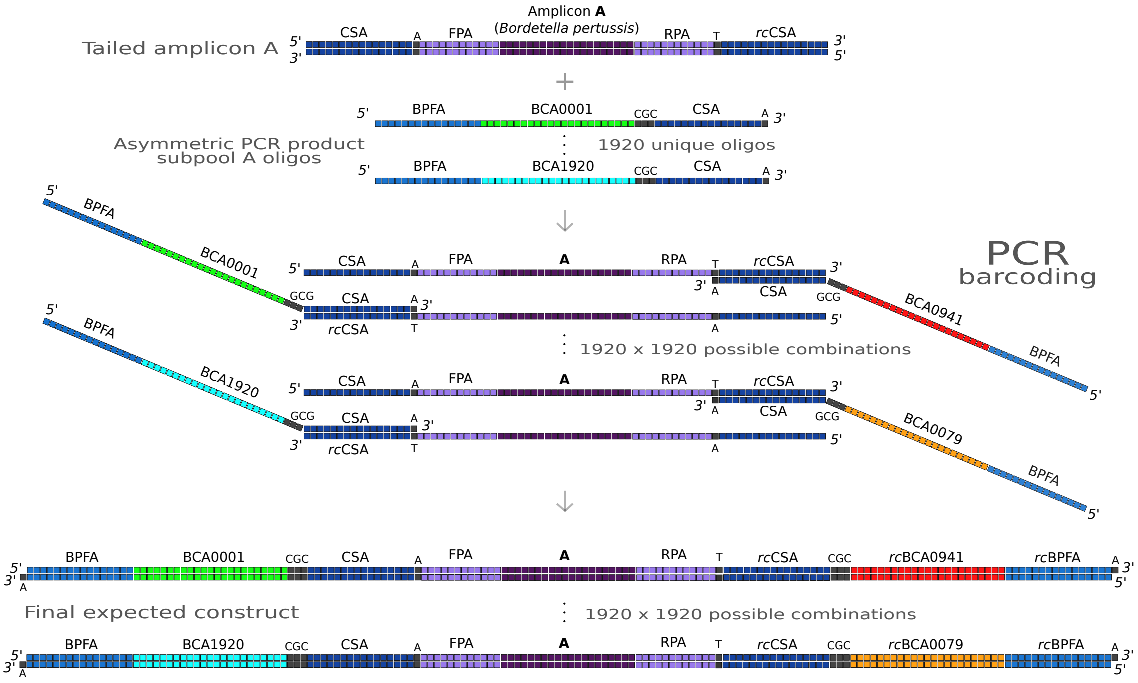

Supplementary Figure 2: PCR barcoding of tailed amplicons. Direct addition of the tailed amplicon sample A (including the forward and reverse primers FPA/RPA, the consensus sequences CSA/*rc*CSA and the A/T supplements) to the ongoing asymmetric PCR reaction of oligo subpool A, allows its random dual PCR barcoding. The same protocol is used for amplicon sample B.

to the remaining subset of $N = C - S$ negative control (unused) barcodes:

$$FAF = \frac{F}{T}\frac{S}{N} \tag{1}$$

where T is the number of demultiplexed reads. In our case, we defined a barcode set with $C = 4096$ members from which a subset comprising $S = 3840$ barcodes was randomly selected and synthesized. In addition, a remaining subset of $N = 256$ barcodes was not synthesized and used as a negative control set. Note that equation **(1)** implicitly assumes that the probability of confusing one barcode for another (a crosstalk event) is the same for any two barcodes, i.e., a uniform probability of confusion between barcodes. Furthermore, it also assumes a uniform concentration of barcodes during molecular tagging. Hence, the average number of reads per barcode affected by crosstalk events in the negative control set $\frac{F}{N}$ should equal, on average, that in the used barcode set. Therefore, in a multiplex sequencing experiment with S barcodes and T reads, we should expect on average $\frac{F}{N} \times S$ reads affected by crosstalk errors, i.e., a crosstalk rate $\frac{F}{T} \times \frac{S}{N}$. In practice, however, the assumptions of equation **(1)** cannot be guaranteed for large barcode sets.

Regarding the assumption of a uniform probability of confusion between barcodes, this implies the design of large barcode sets guaranteeing that the distance between any two barcodes is exactly the same. This is a formidable challenge in Information Theory and, even in the Hamming domain (considering

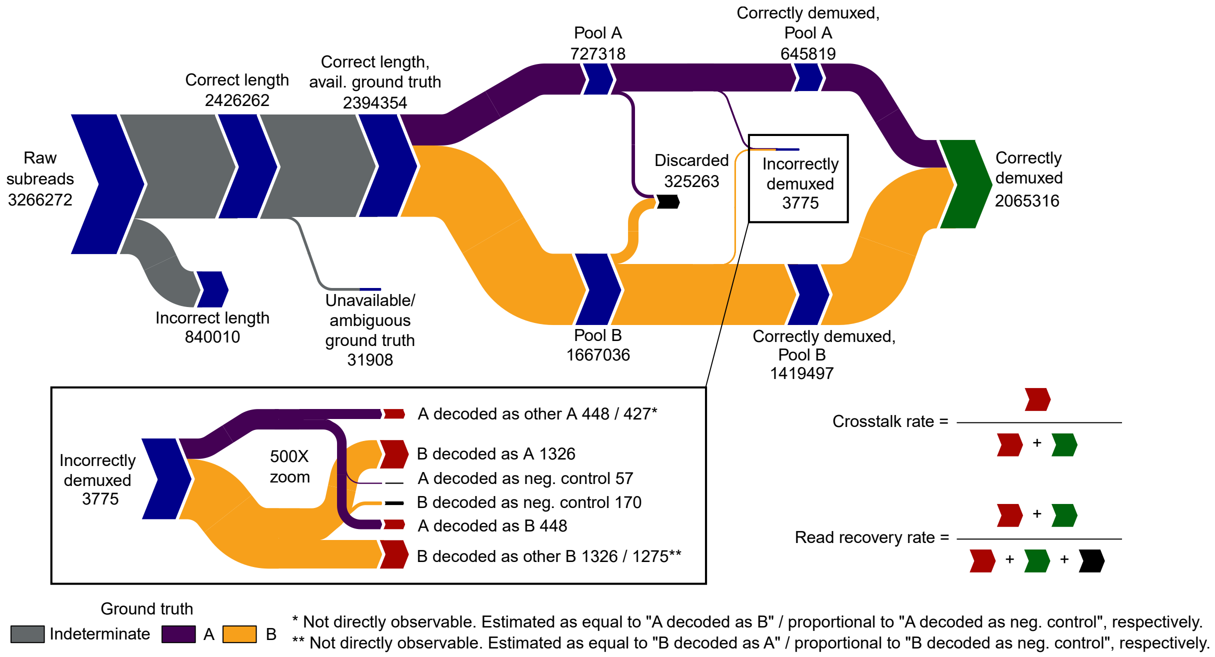

Supplementary Figure 3: A Sankey diagram showing the flow of NS-watermark barcoded subreads (half-reads) until their effective demultiplexing. Subread numbers correspond to the reference threshold point of the demultiplexing performance curve. The initial flow of reads actually involves random-dual barcoded reads whose number is half that of depicted reads until the verification of correct lengths and availability of ground truth molecular identities. **inset** The counts of incorrectly demultiplexed reads are used to compute the read recovery rate and the crosstalk rate as shown.

only substitution errors), has only been achieved for small families of codewords via the so-called perfect codes [2]. No such codes have been described in the Levenshtein (edit distance) domain (considering not only substitutions but also indels). In our case, the histograms of the edit distance between barcodes in each of the oligo subpools and between barcodes in each the oligo subpools and outside them show a high degree of similarity but, as expected, they are not perfectly uniform (**Supplementary Figure 4**). Furthermore, in all cases, we observe the non-negligible presence of pairs of barcodes at relatively low edit distances and thus more prone to be confused for one another.

Regarding the requirement of a uniform concentration of barcodes at the time of molecular tagging, although the oligo pool as a whole can be quantified using standard lab techniques, the concentrations of individual oligos within the pool -which may be different from one another due to variability during the synthesis process- cannot be determined in practice. A reasonable assumption is that these individual concentrations are normally distributed. In this case, because PCR amplification is an exponential process, post-PCR oligo concentrations are expected to follow a log-normal distribution. Indeed, for both oligo subpools used in our experimental work, a log-normal distribution fits the final observed individual barcode frequencies (the closest available proxy for the amplified oligo subpool concentrations) much better, having a long right tail and a sharper fall to zero (**Supplementary Figure 5**). Therefore, in general, the naive assumptions of equation **(1)** will introduce a bias when estimating the crosstalk rate just from the read counts in the set of negative controls. What follows is an effort to quantify and hopefully

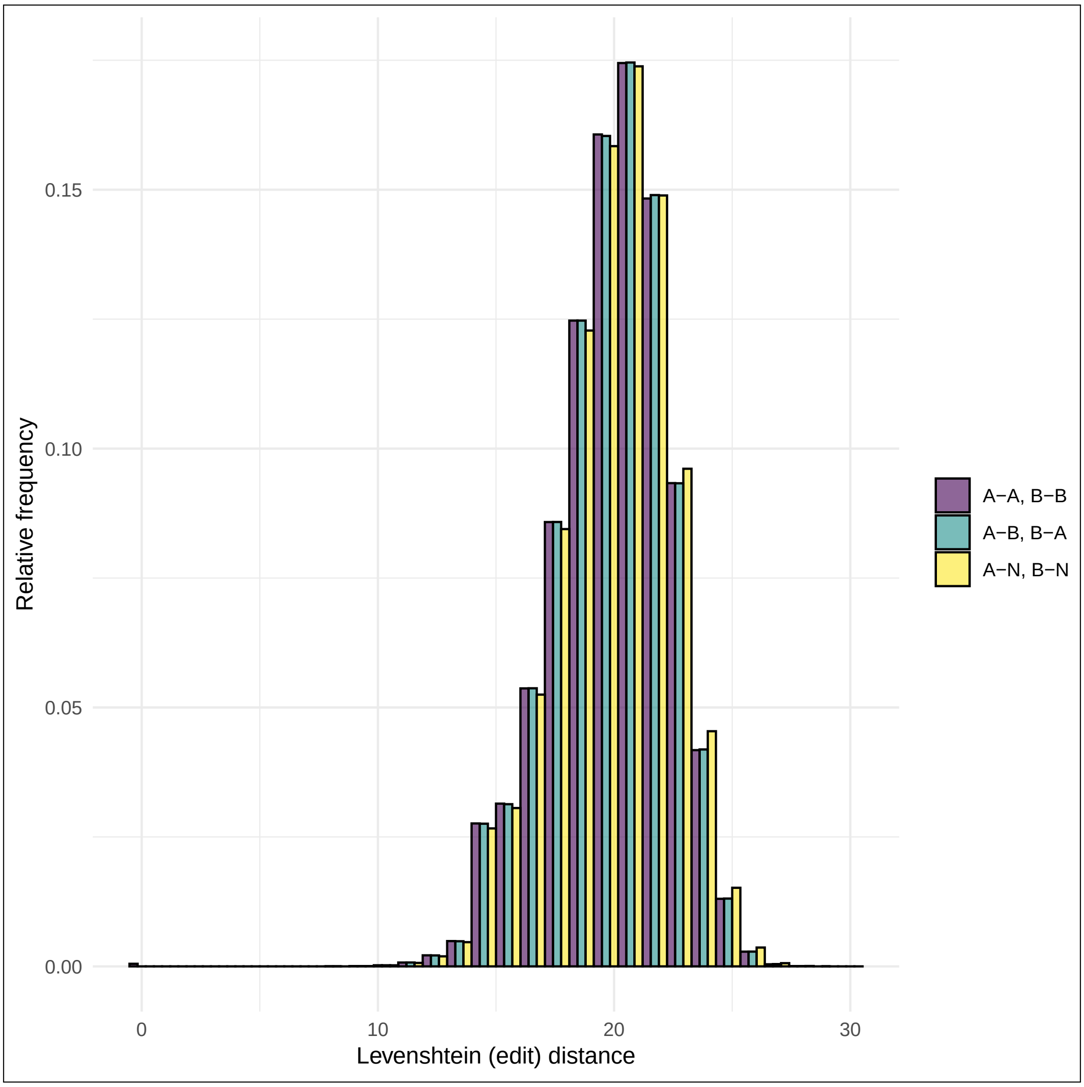

Supplementary Figure 4: Histogram of Levenshtein (edit) distances between the 1920 barcodes in each subpool (A-A, B-B), between the 1920 barcodes in each subpool and the 1920 barcodes in the other subpool (A-B, B-A) and between the 1920 barcodes in each subpool and the 256 barcodes in negative control set (A-N, B-N).

correct this bias.

Using a simple model for crosstalk events, we can assume a crosstalk event occurs when sequencing noise makes a valid barcode "jump" to another valid barcode, where the length of the jump is measured by the edit distance d between the two. Let l be the size in nucleotides of individual barcodes and let p be the overall sequencing error rate. A crosstalk event where a source barcode b_i is confused for a target barcode b_j occurs with probability $p(b_j|b_i)$, where $j = 1, \cdots, C$ and $i = 1, \ldots, S$. Assuming independence between sequencing errors and given that a barcode cannot be confused for itself, we can model $p(b_j|b_i)$ with a zero-truncated binomial distribution [3] with parameters n and p:

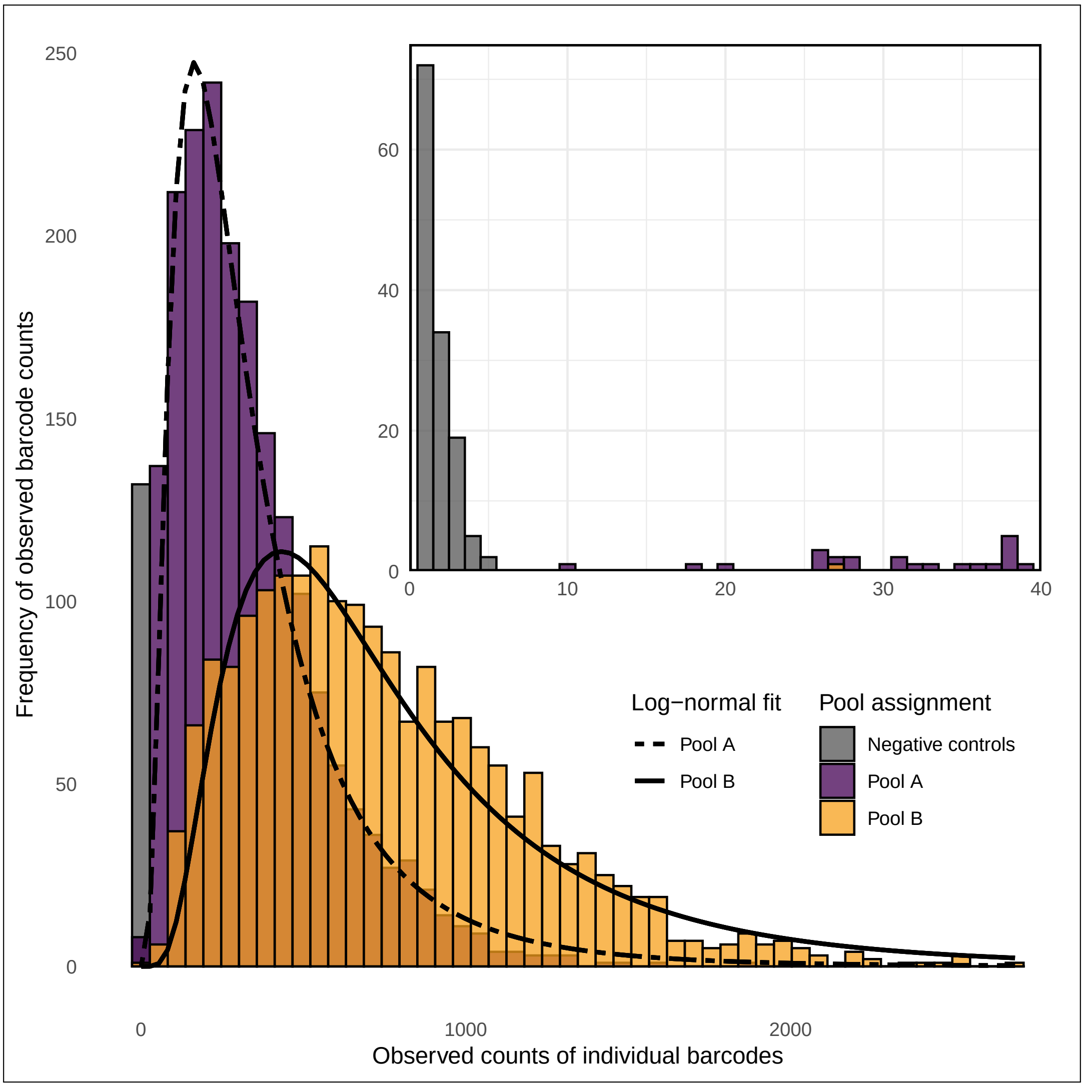

Supplementary Figure 5: After PCR amplification, the relative contribution of individual barcodes in each of the oligo subpools (Pool A and Pool B) fits a log-normal distribution when barcode concentrations are approximated by read counts (the closest available proxy).

$$p(b_j|b_i) = \begin{cases} \frac{\binom{l}{d} p^d (1-p)^{l-d}}{1-(1-p)^l} & j \neq i \\ 0 & j = i \end{cases} \tag{2}$$

where $d \geq 1$ is the pairwise edit distance between barcodes b_i and b_j, where $i = 1, \ldots, S$ and $j = 1, \ldots, C$. Now, let n_t be the total number of sequenced tagged molecules and let $n(b_i)$ be the number of sequenced molecules tagged with barcode b_i, so that $n_t = \sum_{i=1}^{S} n(b_i)$. The expected number of crosstalk events $n_c(b_j)$ to barcode b_j, $j = 1, \ldots, C$, can be computed as follows:

$$n_c(b_j) = \sum_{i=1}^{S} n(b_i)\, p(b_j|b_i) \tag{3}$$

We can compute the expected number of crosstalk events n_c^* as follows:

$$n_c^* = n_{c,1}^* + n_{c,2}^* \quad (4)$$

where $n_{c,1}^*$ is the expected number of crosstalk events in the set of used barcodes, and $n_{c,2}^*$ is the expected number of crosstalk events in the set of unused barcodes (negative controls):

$$n_{c,1}^* = \sum_{j=1}^{S} n_c(b_j) = \sum_{j=1}^{S}\sum_{i=1}^{S} n(b_i)\, p(b_j|b_i) \quad (5)$$

$$n_{c,2}^* = \sum_{j=S+1}^{C} n_c(b_j) = \sum_{j=S+1}^{C}\sum_{i=1}^{S} n(b_i)\, p(b_j|b_i) \quad (6)$$

Provided the crosstalk rate is sufficiently low and all bias is due to amplification (unbiased library preparation and sequencing), we can use the observed read counts $r(b_i)$ for each barcode b_i as a proxy for the number of sequenced molecules $n(b_i)$ tagged with that barcode: $n(b_i) \approx r(b_i)$, $i = 1, \ldots, S$. Therefore, we can approximate the number of crosstalk events in the target and negative control sets as:

$$n_{c,1}^* \approx \sum_{j=1}^{S} n_c(b_j) = \sum_{j=1}^{S}\sum_{i=1}^{S} r(b_i)\, p(b_j|b_i) \quad (7)$$

$$n_{c,2}^* \approx \sum_{j=S+1}^{C} n_c(b_j) = \sum_{j=S+1}^{C}\sum_{i=1}^{S} r(b_i)\, p(b_j|b_i) \quad (8)$$

These read counts can be used to define a bias ratio BR, which measures how far the average crosstalk per barcode in the target barcode set is expected to deviate from that in the negative control set:

$$BR = \frac{\frac{n_{c,1}^*}{S}}{\frac{n_{c,2}^*}{C-S}} \quad (9)$$

For our data ($C = 4096$, $S = 3840$, $l = 36$, $p = 0.1$ and observed values of $r(b_i)$ as per Supplementary Figure 5), BR equals 1.1201, meaning equation **(1)** sub-estimates the crosstalk rate by about 12%. Values of $BR < 1$ are also possible, in which case the error rate as estimated from negative controls would be over-estimating the actual crosstalk rate.

Table S1: List of oligonucleotide sequences

Oligonucleotide Code	Purpose	Sequence (5'-3')
CSA-A-FPA	Tailed forward primer for amplicon A	GCAAGCGGTACACTCAGATCAGACTTCGTCTTCGTGGCCAT
CSA-A-RPA	Tailed reverse primer for amplicon A	GCAAGCGGTACACTCAGATCAGTACAGCGCGCCCGATGCCT
CSB-A-FPB	Tailed forward primer for amplicon B	CAGGAGTTGTCTAGGCGATCACGAGTATCTTCAAGAAAAAGAAATTCAA
CSB-A-RPB	Tailed reverse primer for amplicon B	CAGGAGTTGTCTAGGCGATCACGTGTAAATGCAATTCCTGAGACAT
rcCSB-CGC-**rcBC0000**-rcBPFB	OligoMix sequence, BC0000 (alias BCB0001)	GATCGCCTAGACAACTCCTGCG**CCTGCGAACAATAACAATAACTTGAACTTTCCCGCTG**AGAGACTTACGCTCGACACT
rcCSB-CGC-**rcBC0001**-rcBPFB	OligoMix sequence, BC0001 (alias BCB0002)	GATCGCCTAGACAACTCCTGCG**CCCAATAACAATAACAATACAATCAGCTGCTTATGAC**AGAGACTTACGCTCGACACT
rcCSB-CGC-**rcBC0002**-rcBPFB	OligoMix sequence, BC0002 (alias BCB0003)	GATCGCCTAGACAACTCCTGCG**CAGCGTCACAATAACAATAAGGTGCTGACCGGAGATA**AGAGACTTACGCTCGACACT
rcCSB-CGC-**rcBC0003**-rcBPFB	OligoMix sequence, BC0003 (alias BCB0004)	GATCGCCTAGACAACTCCTGCG**CCTAGTTACAATAACAATAGGTGTACAATACCTAAGG**AGAGACTTACGCTCGACACT
rcCSB-CGC-**rcBC0004**-rcBPFB	OligoMix sequence, BC0004 (alias BCB0005)	GATCGCCTAGACAACTCCTGCG**CAGCCCAACAATAACAATAACCATTATCGGCTCGACA**AGAGACTTACGCTCGACACT
rcCSB-CGC-**rcBC0005**-rcBPFB	OligoMix sequence, BC0005 (alias BCB0006)	GATCGCCTAGACAACTCCTGCG**CGACTTCACAATAACAATAGATTCCGGTGGCCCAGGC**AGAGACTTACGCTCGACACT
rcCSA-GCG-**rcBC0006**-rcBPFA	OligoMix sequence, BC0006 (alias BCA0001)	GATCTGAGTGTACCGCTTGCGC**GCTTTCAACAATAACAATACTAGAAAGGTTAAACGGT**TGAGACAGGAGGTACGTGTT
rcCSB-CGC-**rcBC0007**-rcBPFB	OligoMix sequence, BC0007 (alias BCB0007)	GATCGCCTAGACAACTCCTGCG**CTGGCCCACAATAACAATATATGGGTGACGATGGAGG**AGAGACTTACGCTCGACACT
rcCSA-GCG-**rcBC0008**-rcBPFA	OligoMix sequence, BC0008 (alias BCA0002)	GATCTGAGTGTACCGCTTGCGC**GCATCGCACAATAACAATAACGCCACAAAGAGGCTCC**TGAGACAGGAGGTACGTGTT
rcCSA-GCG-**rcBC0009**-rcBPFA	OligoMix sequence, BC0009 (alias BCA0003)	GATCTGAGTGTACCGCTTGCGC**GGACTATACAATAACAATACAAATCTATGTTTAGCAT**TGAGACAGGAGGTACGTGTT
rcCSA-GCG-**rcBC0010**-rcBPFA	OligoMix sequence, BC0010 (alias BCA0004)	GATCTGAGTGTACCGCTTGCGC**GACGTGGACAATAACAATAGAGACTCTAGCCCCTTAG**TGAGACAGGAGGTACGTGTT
…	…	…
…	…	For brevity, only 20 out of a total of 3840 OligoMix sequences are shown here. For the full list and additional data,
…	…	please see Supplementary File "OligoMix Syntehsized Sequences & Negative controls.ods".
…	…	…
rcCSB-CGC-**rcBC4085**-rcBPFB	OligoMix sequence, BC4085 (alias BCB1915)	GATCGCCTAGACAACTCCTGCG**CGACTTCTAACTGTAACTGGGTGTAACGCACTATGAC**AGAGACTTACGCTCGACACT
rcCSB-CGC-**rcBC4086**-rcBPFB	OligoMix sequence, BC4086 (alias BCB1916)	GATCGCCTAGACAACTCCTGCG**CCTTTCATAACTGTAACTGACTTGACAAAGAGAGATA**AGAGACTTACGCTCGACACT
rcCSA-GCG-**rcBC4087**-rcBPFA	OligoMix sequence, BC4087 (alias BCA1917)	GATCTGAGTGTACCGCTTGCGC**GTGGCCCTAACTGTAACTGCAATCATATGTTCTAAGG**TGAGACAGGAGGTACGTGTT
rcCSB-CGC-**rcBC4088**-rcBPFB	OligoMix sequence, BC4088 (alias BCB1917)	GATCGCCTAGACAACTCCTGCG**CCATCGCTAACTGTAACTGGCTGAGAGGTTAAACCAG**AGAGACTTACGCTCGACACT
rcCSB-CGC-**rcBC4089**-rcBPFB	OligoMix sequence, BC4089 (alias BCB1918)	GATCGCCTAGACAACTCCTGCG**CGACTATTAACTGTAACTGTGACATTGACGAATGTTA**AGAGACTTACGCTCGACACT
rcCSB-CGC-**rcBC4090**-rcBPFB	OligoMix sequence, BC4090 (alias BCB1919)	GATCGCCTAGACAACTCCTGCG**CACGTGGTAACTGTAACTGTGACTCATCGGCATCACG**AGAGACTTACGCTCGACACT
rcCSA-GCG-**rcBC4091**-rcBPFA	OligoMix sequence, BC4091 (alias BCA1918)	GATCTGAGTGTACCGCTTGCGC**GACCAAATAACTGTAACTGATCGTAGGTGGCGGCTGT**TGAGACAGGAGGTACGTGTT
rcCSA-GCG-**rcBC4092**-rcBPFA	OligoMix sequence, BC4092 (alias BCA1919)	GATCTGAGTGTACCGCTTGCGC**GTAGATATAACTGTAACTGGAGACTTGACCGGGCTCC**TGAGACAGGAGGTACGTGTT
rcCSA-GCG-**rcBC4093**-rcBPFA	OligoMix sequence, BC4093 (alias BCA1920)	GATCTGAGTGTACCGCTTGCGC**GTTGAAGTAACTGTAACTGCTTCGGCAATACTAGCAT**TGAGACAGGAGGTACGTGTT
rcCSB-CGC-**rcBC4094**-rcBPFB	OligoMix sequence, BC4094 (alias BCB1920)	GATCGCCTAGACAACTCCTGCG**CTGTGCTTAACTGTAACTGACGCCAACTTTCCCTTAG**AGAGACTTACGCTCGACACT
BPFA	OligoMix PCR Amp. Pool A	AACACGTACCTCCTGTCTCA
rcCSA	OligoMix PCR Amp. Pool A	GATCTGAGTGTACCGCTTGC
BPFB	OligoMix PCR Amp. Pool B	AGTGTCGAGCGTAAGTCTCT
rcCSB	OligoMix PCR Amp. Pool B	GATCGCCTAGACAACTCCTG
BPFA	Asymmetric PCR Pool A	AACACGTACCTCCTGTCTCA
rcCSA-GCG	Asymmetric PCR Pool A	GATCTGAGTGTACCGCTTGCGCG
BPFB	Asymmetric PCR Pool B	AGTGTCGAGCGTAAGTCTCT
rcCSB-CGC	Asymmetric PCR Pool B	GATCGCCTAGACAACTCCTGCGC

References

[1] Matthias Meyer, Udo Stenzel, and Michael Hofreiter. Parallel tagged sequencing on the 454 platform. *Nature Protocols*, 3:267–278, 2008.

[2] F. J. MacWilliams and N. J. A. Sloane. *The theory of error correcting codes / F.J. MacWilliams,*

N.J.A. Sloane. North-Holland Pub. Co. ; sole distributors for the U.S.A. and Canada, Elsevier/North-Holland Amsterdam ; New York : New York, 1977.

[3] D. J. Finney. The truncated binomial distribution. *Ann Eugen*, 14(4):319–328, Jun 1949.
